# Atypical *ATMs*: Broadening the phenotypic spectrum of *ATM*-associated hereditary cancer

**DOI:** 10.3389/fonc.2023.1068110

**Published:** 2023-02-14

**Authors:** Nicholas A. Borja, Rachel Silva-Smith, Marilyn Huang, Dipen J. Parekh, Daniel Sussman, Mustafa Tekin

**Affiliations:** ^1^ Dr. John T. Macdonald Foundation Department of Human Genetics, Miller School of Medicine, University of Miami, Miami, FL, United States; ^2^ Division of Gynecologic Oncology, Sylvester Comprehensive Cancer Center, University of Miami, Miami, FL, United States; ^3^ Desai Sethi Urology Institute, Miller School of Medicine, University of Miami, Miami, FL, United States; ^4^ Division of Digestive Health and Liver Diseases, University of Miami Miller School of Medicine, Miami, FL, United States; ^5^ John P. Hussmann Institute for Human Genomics, Miller School of Medicine, University of Miami, Miami, FL, United States

**Keywords:** ATM, ataxia-telangiectasia mutated (ATM), germline, pathogenic variant (PV), hereditary cancer, cancer susceptibility association

## Abstract

Heterozygous, loss-of-function germline variants in *ATM* have been associated with an increased lifetime risk of breast, pancreas, prostate, stomach, ovarian, colorectal, and melanoma cancers. We conducted a retrospective review of thirty-one unrelated patients found to be heterozygous for a germline pathogenic variant in *ATM* and identified a significant proportion of patients in this cohort with cancers not currently associated with the ATM hereditary cancer syndrome, including carcinomas of the gallbladder, uterus, duodenum, kidney, and lung as well as a vascular sarcoma. A comprehensive review of the literature found 25 relevant studies where 171 individuals with a germline deleterious *ATM* variant have been diagnosed with the same or similar cancers. The combined data from these studies were then used to estimate the prevalence of germline *ATM* pathogenic variants in these cancers, which ranged between 0.45% and 2.2%. Analysis of tumor sequencing performed in large cohorts demonstrated that the frequency of deleterious somatic *ATM* alterations in these atypical cancers equaled or exceeded the alteration frequency in breast cancer and occurred at a significantly higher rate than in other DNA-damage response tumor suppressors, namely *BRCA1* and *CHEK2.* Furthermore, multi-gene analysis of somatic alterations in these atypical cancers demonstrated significant co-occurrence of pathogenic alterations in *ATM* with *BRCA1* and *CHEK2*, while there was significant mutual exclusivity between pathogenic alterations in *ATM* and *TP53.* This indicates that germline *ATM* pathogenic variants may play a role in cancer initiation and progression in these atypical *ATM* malignancies, potentially influencing these cancers to be driven toward DNA-damage repair deficiency and away from loss of *TP53*. As such, these findings provide evidence for broadening of the *ATM*-cancer susceptibility syndrome phenotype to improve the recognition of affected patients and provide more efficacious, germline-directed therapies.

## Introduction

*ATM* (OMIM 607585) encodes the PI3K-related serine/threonine protein kinase comprising 3,056 amino acids, ataxia-telangiectasia mutated. This protein kinase primarily resides in the nucleus of dividing cells and has long been recognized as a key upstream modulator of the response to DNA double-stranded breaks, as well as oxidative and other genotoxic stresses. A critical function of ATM involves the recruitment and cooperation with DNA-damage sensing proteins such as BRCA1 in the face of double-strand breaks (1). ATM also plays a critical role in the DNA damage response through the phosphorylation of downstream substrates including CHK2 and p53, which then stimulate the cell-cycle checkpoint arrest and cellular apoptosis pathways, respectively (1, 2).

Biallelic pathogenic loss-of-function variants in *ATM* have long been associated with the genomic instability syndrome, ataxia-telangiectasia. Manifestations of the disorder include cerebellar ataxia, oculocutaneous telangiectasia, immunodeficiency, radiosensitivity, premature aging, and a predisposition to cancer development, primarily of lymphoid origin (3).

It was later recognized that women who are heterozygous for pathogenic loss-of-function variants in *ATM* have an increased risk for breast cancer, leading *ATM* to become regarded as a moderate penetrance breast cancer susceptibility gene, conferring a 2.3-fold increased risk for breast cancer compared to the general population (4, 5).

The risk of cancer among individuals heterozygous for *ATM* pathogenic variants has since been demonstrated for a broader range of malignancies. *ATM* pathogenic variant carriers appear to be at moderate-to-high increased risk for pancreatic, prostate, and gastric cancers and at low-to-moderate increased risk for ovarian and colorectal cancer as well as melanoma (6–9).

The association of *ATM* with cancer susceptibility has directly influenced clinical practice, from the creation of gene panels for molecular testing to cancer surveillance guidance and treatment recommendations. Here we present six novel cases of individuals with carcinomas of the gallbladder, uterus, duodenum, kidney, and lung as well as a sarcoma, all harboring germline *ATM* pathogenic variants, which suggests further expansion of the *ATM*-associated cancer susceptibility phenotype.

## Methods

### Review of patients harboring deleterious germline *ATM* variants

We retrospectively reviewed patients seen in our hereditary cancer clinic between March 2017 and December 2021 who had undergone germline testing with next-generation, multi-gene sequencing. We then identified all patients found to harbor germline likely pathogenic or pathogenic variants in *ATM* producing a loss-of-function using the criteria of the American College of Medical Genetics standards and guidelines for sequence variant interpretation (10). A comprehensive chart review through the electronic medical record was conducted to collect relevant clinical data including patient demographics, personal and family cancer history, as well as complete genetic testing results. Evidence substantiating the pathogenicity of the germline *ATM* variants among the six patients with atypical *ATM*-associated cancers was collected (**Supplementary Table 1**).

Multi-gene hereditary cancer syndrome testing was performed in commercial clinical laboratories accredited by the College of American Pathologists and certifyed by the Clinical Laboratory Improvement Amendment. Laboratories used included Ambry Genetics (Aliso Viejo, CA), GeneDx (Gaithersburg, MD), Myriad (Salt Lake City, UT), Sema4 (Stamford, CT), or Invitae (San Francisco, CA). One patient had sequencing performed at the medical institution where he had previously received care (Memorial Sloan Kettering, New York, NY). The number of analyzed genes ranged from 25-84, each of which was associated with hereditary cancers in peer-reviewed scientific literature (**Supplementary Table 2**).

### Literature search of reported deleterious germline *ATM* variants in atypical cancers

A comprehensive review of the peer-reviewed literature through MEDLINE was conducted using PubMed for articles published between 1970 and July 2022. Search keywords included “pathogenic,” “germline,” “ATM,” “variant,” “hereditary,” “cancer,” “gallbladder,” “uterus,” “duodenum,” “kidney,” “lung,” “epithelioid hemangioendothelioma,” and then later expanded to include “biliary tract,” “small bowel,” “ampulla,” and “sarcoma.” Keywords were connected by the Boolean functions AND and OR.

All cross-sectional studies involving thirty or more patients affected with cancer of the biliary tract, uterus, small intestine/ampulla, kidney, lung, or sarcoma, who had undergone hereditary cancer predisposition testing with a multigene panel, exome, or genome sequencing were considered for inclusion. Studies using redundant patient information drawn from large datasets were excluded from the review. The study text and supplemental information were reviewed for patient characteristics, total germline pathogenic variants detected, and specific germline *ATM* variants identified.

The prevalence for each cancer type was estimated by dividing the number of individuals with a germline *ATM* pathogenic variant by the total sample size. To estimate the prevalence among those with hereditary cancer susceptibility, the number of individuals with a germline *ATM* pathogenic variant was divided by the number of those testing positive for any pathogenic variant in a hereditary cancer gene.

### Obtaining frequency of somatic alterations in *ATM* for atypical cancers

De-identified genomic sequencing data from The Cancer Genome Atlas and other large-scale, cancer-specific sequencing studies were accessed and queried online through the cBioPortal for Cancer Genomics at https://www.cbioportal.org/ (11, 12). Patient cohorts were primarily selected from the TCGA pan-cancer atlas, and the accession numbers for each tumor-specific analysis were recorded (**Supplementary Methods**). Somatic alterations included were loss-of-function single nucleotide variants (SNVs), indels, and copy number variants (CNVs) classified as pathogenic or likely pathogenic. Statistical analysis to compare deleterious alteration counts of *ATM*, *BRCA1*, and *CHEK2* within each tumor type was performed with Microsoft Excel version 16.64 (Microsoft, WA, USA) using the Z-test for independent proportions. A p-value equal to or less than 0.05 was considered significant.

### Determining mutual exclusivity and co-occurrence among *ATM* and other DNA-damage response genes

Pathogenic alterations in the relevant cancer types accessed through cBioPortal as above were jointly analyzed to determine the relationship between variants in *ATM, BRCA1, CHEK2*, and *TP53*. A Log2 odds ratio was used to calculate how strongly the presence or absence of alterations in one gene was associated with the presence or absence of alterations in a second gene within the selected samples. A q-value derived from the Benjamini-Hochberg FDR correction procedure equal to or less than 0.05 was considered significant.

### Compliance with ethical standards

All studies involving human participants were approved by the University of Miami institutional research board (IRB #20081166) and are in accordance with the 1964 Helsinki declaration and its later amendments or comparable ethical standards.

## Results

**Figure 1:**
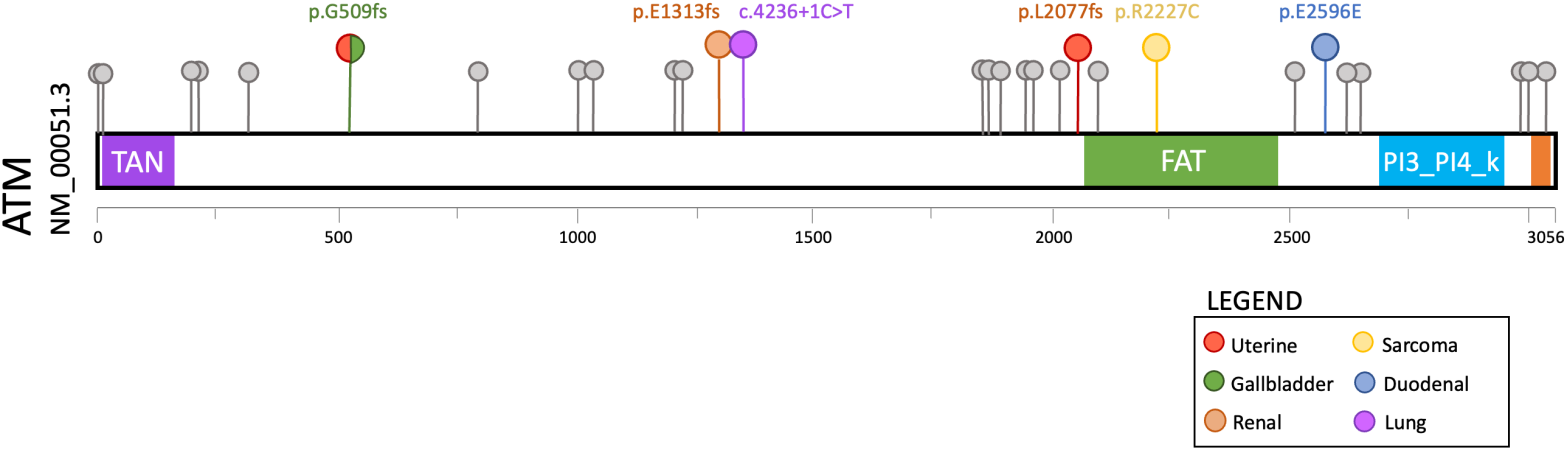
Lollipop plot of germline *ATM* pathogenic variants detected in 31 consecutive patients with a personal or family history of cancer who underwent hereditary cancer susceptibility testing. The six variants associated with cancers that are atypical for *ATM* hereditary cancer susceptibility are highlighted in color (see legend).

We reviewed the patients seen in our hereditary cancer clinic who had undergone comprehensive multigene panel testing and were found to be heterozygous for a germline pathogenic variant in *ATM*. A total of thirty-one patients met these criteria, none of which had a pathogenic variant in any other hereditary cancer gene. Twenty-five of these patients had a personal or family history of cancer that is consistent with the currently described spectrum of *ATM*-associated malignancies. Notably, the remaining six patients (19.4%) had a personal history of cancers not currently associated with *ATM* hereditary cancer susceptibility. Only one of these six patients was identified to harbor variants of uncertain significance in other hereditary cancer genes (**Supplementary Table 3**). To evaluate for genotype-phenotype correlations, we compared the pathogenic *ATM* variants in the 6 atypical cancer cases to the 25 typical cancer cases, though no clear differences were evident (**Figure 1**).

The personal and family history of cancer among the six patients with malignancies atypical for *ATM*-associated hereditary cancer susceptibility were then evaluated in detail (**Table 1**). We observed that two of the six patients had a personal history of known *ATM*-associated cancers. Patient 1 had a history of breast adenocarcinoma, and Patient 6 had a history of melanoma. All six patients had at least one 1^st^ or 2nd-degree relative with *ATM*-associated cancer including breast, pancreatic, prostate, gastric, and colorectal adenocarcinomas. Four of the six patients meet current NCCN criteria for germline genetic testing based on either their personal history of cancer or family history of cancer (**Supplementary Table 4**).

**Table 1:** Clinical summary of the six patients identified to harbor a pathogenic germline variant in *ATM* and a personal history of cancer not associated with *ATM* hereditary cancer syndrome.

Patient	Ethnicity	Cancer History (age at diagnosis)	Germline *ATM* variants	Family History of Cancer Among 1^st^ and 2^nd^ Degree Relatives
Patient 1	Italian & Portuguese	*Breast adenocarcinoma* (50s) Gallbladder carcinoma in situ (50s) Uterine endometroid carcinoma (60s)	c.1542delT p.Gly509Glufs*3	Mother: *breast cancer* (60s), *pancreatic cancer* (70s) Father: renal cancer (80s) Maternal aunt: *breast cancer* (60s), gallbladder cancer (70s) Paternal uncle: *colorectal cancer* (60s) Paternal grandfather: *colon cancer* (70s), *gastric cancer* (70s)
Patient 2	Ashkenazi Jewish	Uterine serous carcinoma (60s)	c.6228del p.Leu2077Phefs*5	Brother: *colon cancer* (60s) Father: *prostate cancer* (80s) Paternal uncle: *gastric cancer* (70s) Maternal aunt: bladder cancer (80s)
Patient 3	Lebanese	Duodenal adenocarcinoma (60s)	c.7788G>A p.Glu2596Glu	Brother: *prostate cancer* (50s) Mother: *breast cancer* (70s) Maternal aunt: *breast cancer* (70s) Maternal aunt: *breast cancer* (20s)
Patient 4	Ashkenazi Jewish	Clear cell renal cell carcinoma (40s)	c.3935dupG p.Glu1313Argfs*8	Mother: *rectal cancer* (70s) Maternal uncle: leukemia (70s) Maternal grandfather: leukemia (40s)
Patient 5	Caucasian	Epithelioid hemangioendothelioma of lung (40s)	c.6679C>T p.Arg2227Cys	Father: brain cancer (20s) Maternal aunt: *breast cancer* (40s), throat cancer (60s) Maternal aunt: *colon cancer* (70s) Maternal aunt: *colon cancer* (60s) Maternal aunt: *colon cancer* (70s) Paternal uncle: *melanoma* (50s)
Patient 6	Ashkenazi Jewish	*Melanoma* (10s) Lung adenocarcinoma (60s)	c.4236+1G>T	Father: *prostate cancer* (70s) Paternal grandfather: *gastric cancer* (50s) Nephew: anaplastic astrocytoma (20s) Maternal grandmother: *breast cancer* (70s)

*Italicized cancers* are those currently associated with *ATM* hereditary cancer predisposition syndrome.

### Comprehensive review of atypical *ATM*-cancer associations

To better characterize the association between germline *ATM* pathogenic variants and the atypical cancers seen in our six patients, we performed a comprehensive review of all cross-sectional studies where germline sequencing was performed in patients diagnosed with carcinomas of the biliary tract, uterus, small bowel and ampulla, kidney, and lung, as well as sarcomas. We identified twenty-five broadly relevant, cross-sectional studies where next-generation sequencing of cancer susceptibility genes was performed (**Supplementary Table 5**, 13–37). A total of 171 unique patients harboring a germline pathogenic variant in *ATM* and diagnosed with one of the atypical *ATM* cancers were identified. Of these, we found that fewer than 20 cases of germline *ATM* pathogenic variants have been associated with biliary tract carcinoma, uterine carcinoma, small bowel carcinoma, or sarcoma, while we identified 95 cases of lung adenocarcinoma and 32 cases of renal cell carcinoma associated with germline *ATM* pathogenic variants.

**Figure 2:**
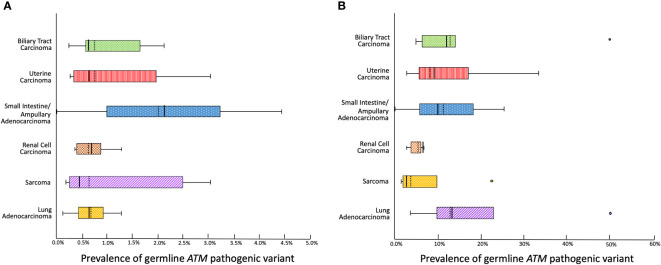
Box and whiskers plot depicting **(A)** The prevalence of a germline pathogenic *ATM* variant among individuals affected with each cancer type based on pooled data from a comprehensive review of cross-sectional studies. **(B)** The prevalence of a germline pathogenic *ATM* variant among individuals affected with each cancer who test positive for hereditary cancer predisposition (mean depicted with a solid vertical line, median depicted with a dashed vertical line).

We then estimated the prevalence of germline *ATM* pathogenic variants for each relevant cancer type by compiling data from each reviewed study (**Figure 2A**). The prevalence was calculated to be as low as 0.45% for sarcoma, 0.57% for uterine carcinoma, 0.6% for biliary tract carcinoma, 0.63% for lung carcinoma, and 0.87% for renal cell carcinoma. In the case of small intestine & ampullary carcinoma, the prevalence reached 2.2% albeit with a broad range due to the limited sample size. We then calculated the prevalence of germline *ATM* pathogenic variants among individuals diagnosed with an atypical *ATM* cancer who tested positive for hereditary cancer predisposition (**Figure 2B**). We found that among sarcoma and renal cell carcinoma patients diagnosed with a hereditary cancer susceptibility syndrome, the prevalence of a germline *ATM* pathogenic variant was 2.4% and 6.5%, respectively. Meanwhile, patients with biliary tract, uterine, small bowel & ampullary, as well as lung carcinomas who tested positive for a hereditary cancer susceptibility syndrome had a germline *ATM* pathogenic variant in 9% - 12.7% of cases.

These data provide evidence that germline *ATM* pathogenic variants exhibit a low penetrance for the atypical cancers investigated herein. Nevertheless, these germline *ATM* pathogenic variants were associated with a significant proportion of the known causes for hereditary cancer susceptibility in these cancers.

### Somatic alterations in *ATM* detected in tumor sequencing databases across the investigated cancer types

Next, we explored available tumor sequencing data from large cohorts that closely match the tumor type and subtype described in our patient cases with the aim of further clarifying the biological relevance of *ATM* loss-of-function variants in the carcinogenesis of gallbladder, uterus, duodenum, kidney, lung, and sarcomas. We queried data available through cBioPortal and determined the frequency of somatic deleterious *ATM* variants and compared this alteration frequency to that of *BRCA1* and *CHEK2*, as these are DNA-damage response tumor suppressors also implicated in hereditary cancer, but not established to drive oncogenesis in the cancer types being investigated.

**Figure 3:**
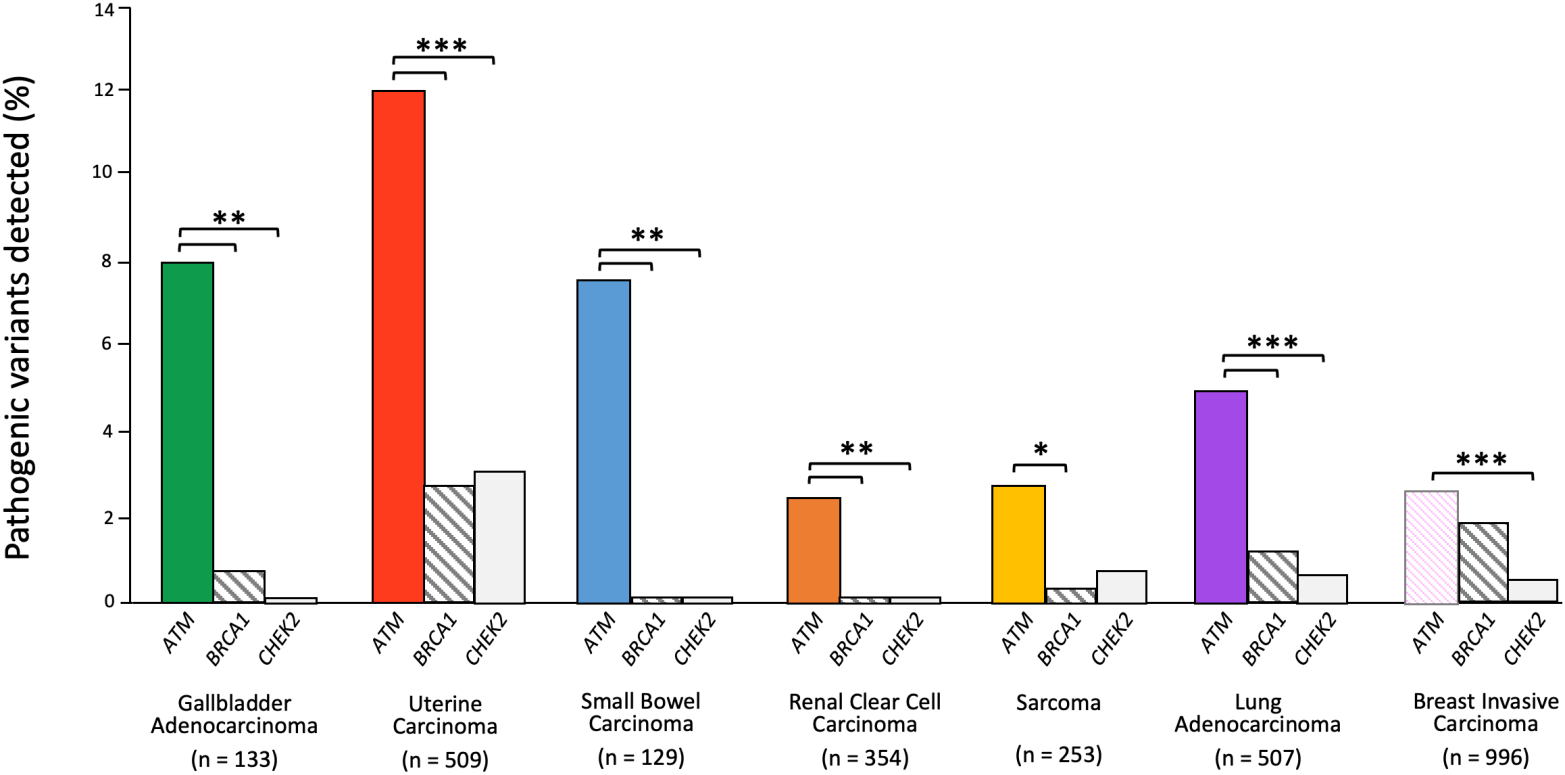
*ATM* somatic alteration frequency compared to *BRCA1* and *CHEK2* alterations in relevant cancers. Z-test for independent proportions was used to identify significant differences (* p ≤ 0.05, ** p ≤ 0.01, *** p ≤ 0.001, all other comparisons not significant). Tumor sequencing data was accessed through cBioPortal.

*ATM* variants were detected in as many as 12% of uterine carcinomas, 8% of gallbladder carcinomas, 7.8% of small bowel carcinomas, and 5% of lung adenocarcinomas, whereas *BRCA1* and *CHEK2* variants were found in less than 3% of uterine carcinomas, less than 2% of lung adenocarcinomas, less than 1% of gallbladder carcinomas, and none were found in small bowel carcinomas (**Figure 3**). Clear cell renal cancer had the fewest *ATM* pathogenic alterations, reported at a frequency of 2.3%, though still at a significantly higher frequency than that of *BRCA1* and *CHEK2*. In the case of sarcoma, the *ATM* alteration frequency was 2.8%, which was significantly greater than *BRCA1* but not *CHEK2*. We then compared these alteration frequencies to those seen in breast cancer, where all three genes have been implicated in carcinogenesis, and found pathogenic variants in *ATM* only occurred in 2.5% of cases and with a similar frequency to *BRCA1*, though more frequently than *CHEK2*. These data provide complementary evidence that *ATM* may contribute to cancer risk in each of these cancer types.

**Figure 4:**
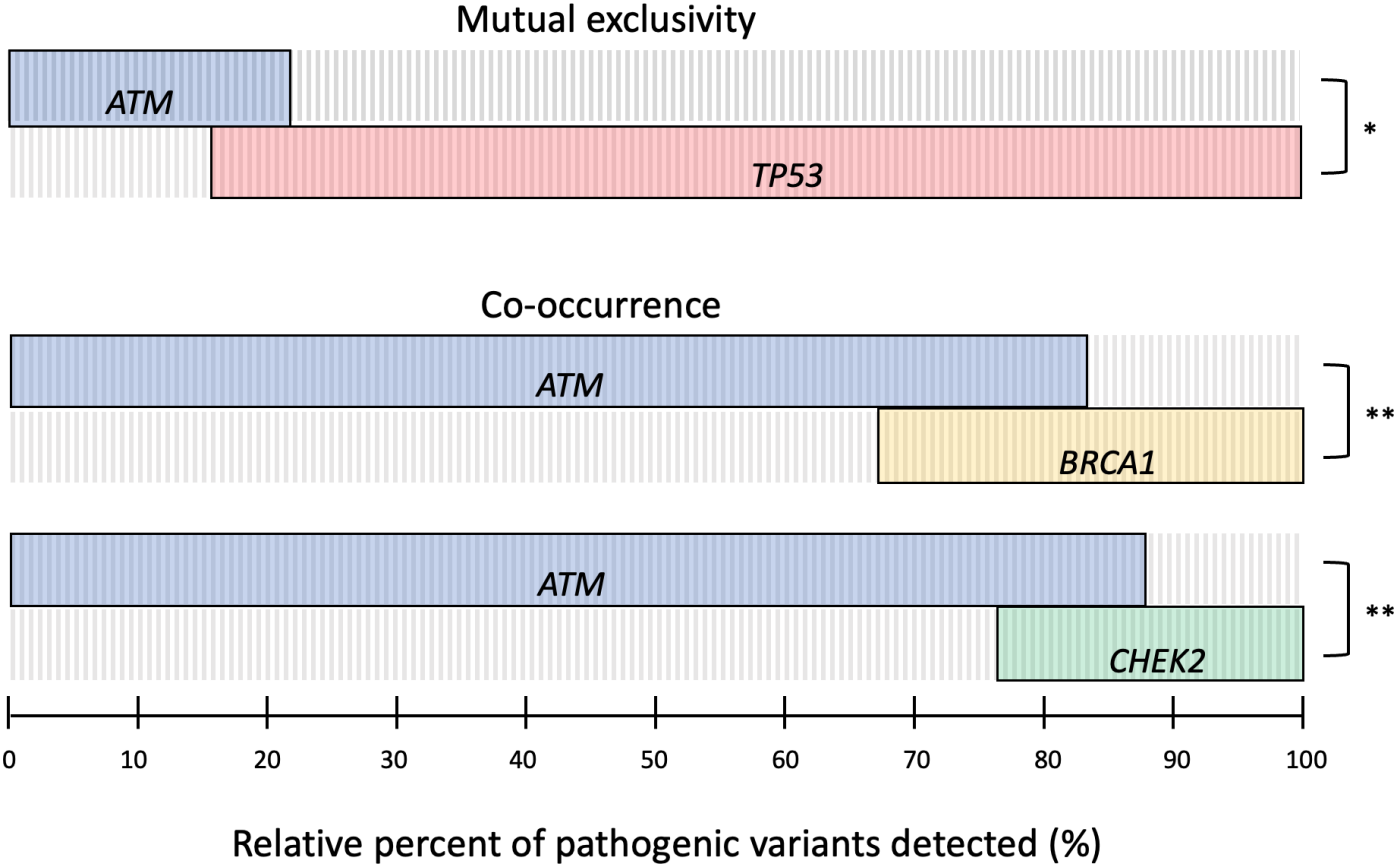
Oncoprint plot of somatic pathogenic alterations in *ATM, TP53, BRCA*, and *CHEK2* across relevant tumor sequencing data to determine the relationship between alterations. Log2 odds ratio was used to identify mutual exclusivity and co-occurrence, with significant differences defined by Benjamini-Hochberg FDR correction (* q ≤ 0.05, ** q ≤ 0.01, *** q ≤ 0.001).

To better understand the influence of germline and somatic *ATM* variants on carcinogenesis in these atypical *ATM* cancers, we pooled the cancer sequencing data to examine the relationship between *ATM* alterations and the alteration of other genes in the DNA damage response and cell cycle pathways (**Figure 4**). We found a significant co-occurrence of pathogenic alterations in *ATM* with pathogenic alterations in *BRCA1* and *CHEK2* (Log2 OR 2.7, 2.5, p<0.01). In addition, we saw significant mutual exclusivity between *ATM* and *TP53* (Log2 OR -0.67, p<0.05). This suggests that the presence of germline *ATM* pathogenic variants may influence associated cancers to be driven toward DNA-damage repair deficiency, and away from loss of *TP53*, the most ubiquitous driver of cancer (38).

## Discussion

Here we describe six unrelated patients found to be heterozygous for germline, loss-of-function variants in *ATM* and diagnosed with at least one malignancy that is not currently associated with the *ATM* cancer susceptibility syndrome, including gallbladder carcinoma, uterine carcinoma, renal cell carcinoma, duodenal adenocarcinoma, lung adenocarcinoma, and a vascular sarcoma. These atypical cancers were diagnosed in a significant proportion of patients with *ATM*-associated hereditary cancer susceptibility in our cohort. Nevertheless, our review of the literature indicates that the prevalence of germline *ATM* pathogenic variants among individuals diagnosed with these atypical cancers appears to be less than 1% in most cases.

Exploration of somatic alterations in The Cancer Genome Atlas (TCGA) datasets demonstrated there was a high frequency of pathogenic alterations in *ATM* relative to other tumor suppressors associated with hereditary cancer syndromes, suggesting that *ATM* loss may contribute to oncogenesis within these cancer types. Furthermore, our analysis reveals that alterations in *ATM* tend to co-occur with pathogenic alterations in *BRCA1* and *CHEK2* while being mutually exclusive of *TP53*. This latter finding underscores the shared functional pathway between *ATM* and *TP53* and reinforces prior work showing that DNA damage creates selection pressure for the inactivation of p53 that can be abrogated through the loss of *ATM* (39, 40). More broadly, these pathway interactions illustrate how germline *ATM* pathogenic variants may influence the acquisition of somatic alterations throughout cancer initiation and progression.

The cases reported in this series exemplify how the tumor spectra associated with cancer susceptibility syndromes are broadening through the clinical use of pan-cancer panels. The germline pathogenic variants in *ATM* detected in all six of our patients were discovered through comprehensive hereditary cancer panels. This testing approach is supported by studies demonstrating significantly higher detection rates compared to panels oriented specifically to the genes that are most relevant to the personal or family history of cancer (41–43). The heterogeneity in cancers documented among our patients with germline *ATM* variants and their families further emphasizes the value of pan-cancer panels, and certainly the importance of including *ATM* in multi-gene panels testing for hereditary cancer predisposition.

Moreover, our cases illustrate the way NCCN Guidelines may significantly limit the detection of hereditary cancer susceptibility, as many cancer types, particularly those with a low prevalence, do not currently qualify individuals for genetic testing. In our series, neither Patient 4 nor Patient 6 met guidelines for hereditary cancer testing based on their personal or family history of cancer, illustrating circumstances in which *ATM* germline variants may go undetected. This was underscored in a recent study where germline genetic testing in an unselected pan-cancer patient population found that over half of the patients who tested positive for pathogenic variants were not eligible for hereditary cancer testing based on current guidelines (44). Improving the detection of *ATM*-associated hereditary cancer is clinically meaningful as it can enable cascade genetic testing for family members who are at risk, facilitate appropriate cancer surveillance for those affected, and prompt the use of precision-based therapies.

Although no specific *ATM* genotype-phenotype correlations were discerned in this study, we did observe that the *ATM* c.1564_1565delGA variant was present in two patients with uterine cancer, and the *ATM* p.V2716A variant was present in two patients with lung adenocarcinoma. In addition, we found the germline *ATM* variant c.6679C>T identified in Patient 5 with epithelioid hemangioendothelioma was also seen in a case of endometrioid uterine cancer, and the germline *ATM* variant c.4236+1G seen in Patient 6 with lung adenocarcinoma was also present in a case of renal cell carcinoma. Meanwhile, none of the pathogenic germline *ATM* variants identified in our six patients overlapped among the somatic loss-of-function *ATM* variants present in the cancer sequencing datasets. Larger sample sizes will be needed to systematically investigate whether specific germline *ATM* variants confer a greater risk for atypical *ATM* cancers.

A limitation of this study is that we cannot exclude the possibility that our patients’ malignancies were incidental to, rather than caused by the pathogenic variant in *ATM*, as the incidence of heterozygosity for pathogenic variants in *ATM* has been estimated in gnomAD for European populations to be 0.48% (45). A segregation analysis of our patients’ families may have strengthened the association between atypical cancers and pathogenic variants in *ATM*, as this would have revealed whether the gallbladder carcinoma in the maternal aunt of Patient 1, the leukemias in the maternal uncle and grandfather of Patient 4, or the anaplastic astrocytoma in the paternal nephew of Patient 6 were also associated with pathogenic variants in *ATM*. Moreover, we were not able to confirm whether *ATM* loss of heterozygosity occurred in our six patients, which could have provided further evidence that *ATM* is driving carcinogenesis based on the two-hit hypothesis (46). Yet, this does not appear to be a reliable finding as the frequency of biallelic two-hit events across all cancers where a germline pathogenic variant is present has been estimated at 38.3%, with a recent study finding that *ATM* somatic second hits occurred in 11.3% in a wide spectrum of cancers including breast, pancreatic, bladder, uterine and lung cancers, among others (15, 47). A case-control analysis for each of the atypical-*ATM* cancer types could have also provided compelling evidence of an association. This has been previously performed in two cohorts of patients with lung adenocarcinoma, demonstrating a significant enrichment for germline pathogenic variants in *ATM* in cases compared to controls (48, 49). Unfortunately, the feasibility of case-control studies is limited by the rarity of many of these cancers, as well as the low penetrance of *ATM* in association with these atypical cancers.

Nevertheless, our finding of relatively frequent somatic *ATM* alterations across many of the atypical cancer types has been corroborated by an analysis showing that *ATM* was among the most frequently mutated genes in the DNA-damage response pathway across 33 distinct types of cancers (50). This evidence has also been bolstered by a recent study demonstrating a high prevalence of germline pathogenic variants in *ATM* relative to other hereditary cancer genes across a spectrum of cancers lacking testing guidelines, including bladder, brain, esophagus, and head and neck cancers (15). Furthermore, our identification that pathogenic *ATM* alterations in these atypical cancers are associated with the co-occurrence of alterations in *BRCA1* and *CHEK2*, and mutual exclusivity with *TP53*, suggests that cancers with a germline pathogenic variant in *ATM* may depend on specific molecular alterations that confer targetable vulnerabilities. This has already been documented in patients with metastatic prostate cancer harboring a germline pathogenic variant in *ATM* sensitizing to PARP inhibition, as well as in patients with pancreatic cancer where monoallelic pathogenic variants in *ATM* produce susceptibility to combined therapies that synergistically target the DNA-damage response pathway (51, 52).

In conclusion, this study combines detailed clinical phenotyping with a comprehensive review of relevant germline *ATM* reports and large-scale tumor sequencing data to propose that germline pathogenic variants in *ATM* may be associated with the development of cancers of the gallbladder, duodenum, uterus, kidney, and lung as well as sarcoma cancers. Consideration should be given to a broadening of the *ATM*-cancer susceptibility syndrome phenotype within hereditary cancer testing guidelines, as this has the potential to improve the detection of affected patients and facilitate the use of more effective cancer treatments.

## Data availability statement

The datasets presented in this study can be found in online repositories. The names of the repository/repositories and accession number(s) can be found in the article/**Supplementary Material**.

## Ethics statement

The studies involving human participants were reviewed and approved by University of Miami institutional research board (IRB #20081166). Written informed consent for participation was not required for this study in accordance with the national legislation and the institutional requirements.

## Author contributions

NB: Conceptualization, methodology, data curation, formal analysis, project administration, visualization, writing–original draft, and writing–review and editing. RS-S: Conceptualization, investigation, resources, data curation, writing–review and editing. MH: Investigation, resources, data interpretation, writing–review and editing. DP: Investigation, resources, and writing–review and editing. DS: Investigation, resources, writing–review and editing. MT: Conceptualization, methodology, formal analysis, investigation, writing–original draft, and writing–review and editing, visualization, supervision. All authors contributed to the article and approved the submitted version.
