# Supplementary material for: Atypical ATMs: Broadening the phenotypic spectrum of ATM-associated hereditary cancer
Source: Front Oncol. 2023 Feb 14;13:1068110. doi: 10.3389/fonc.2023.1068110 (PMC9971806; doi:10.3389/fonc.2023.1068110)
Supplement: Supplementary file 1 [file DataSheet_1.pdf]

SUPPLEMENTARY TABLE 1. Characterization of the pathogenicity of germline *ATM* variants identified among the patients in the case series

Patients	Evidence of germline *ATM* variant pathogenicity
Patient 1	The patient was found to be heterozygous for a variant in *ATM* c.1524delT, which results in a deletion of one nucleotide at position 1524, causing a translational frameshift with a predicted alternate stop codon (p.G509Efs*3). This alteration is classified as pathogenic as it is expected to result in a loss of function due to premature protein truncation and has been reported in an individual with ataxia-telangiectasia harboring a second pathogenic variant in *ATM* (1).
Patient 2	The patient was found to heterozygous for a variant in *ATM* c.6228delT, which is predicted to result in a premature truncation of the ATM protein at the amino acid position 2081 (p.Leu2077Phefs*5). This *ATM* variant c.6228delT has been reported in an individual with history of breast and pancreatic cancer as well as in a patient with ataxia-telangiectasia and therefore classified as pathogenic (2,3).
Patient 3	The patient was identified to harbor a synonymous variant in *ATM* c.7788G>A (p.Glu2596Glu). Although this preserves a glutamic acid at codon 2596, this base pair substitution disrupts the splice donor site, resulting in skipping of exon 52, which codes for a significant portion of the FAT functional domain that maintains the ATM protein stability (4). Moreover, this variant has been reported in patients affected with ataxia-telangiectasia, and as such, it is classified as pathogenic (5,6).
Patient 4	The patient was identified to have the pathogenic variant in *ATM* c.3935dupG, which is predicted to lead to a frameshift and premature translational stop signal at residue 1320 (p.Glu1313Argfs*8). This *ATM* variant has been submitted to the ClinVar database four times since 2016 (ID 232284) in association with ataxia-telangiectasia and hereditary cancer-predisposing syndrome. All four submissions interpret the variant as pathogenic or likely pathogenic.
Patient 5	The patient was found to have a pathogenic variant in *ATM* c.6679C>T, which is predicted to lead to a substitution of the amino acid Arginine for Cysteine at residue 2227, located within the PIK-related kinase, FAT domain. The variant has been shown to reduce *ATM* protein levels as well as *ATM* kinase activity in functional studies and is found in multiple individuals in the compound heterozygous state and affected with ataxia-telangiectasia (7,8). This *ATM* variant has twelve submissions to the ClinVar database (ID 181981) since 2015 in association with ataxia-telangiectasia and hereditary cancer-predisposing syndrome, all of which classify the variant as pathogenic.
Patient 6	The patient is heterozygous for a likely pathogenic variant in *ATM* c.4236+1G>T, which is predicted to disrupt the donor splice site of intron 28 and lead to an absent or disrupted protein product. The variant has been reported in an individual diagnosed with ovarian cancer (9). Moreover, the *ATM* variant has four submissions to the ClinVar database (ID 233837), all of which classify the variant as likely pathogenic.

SUPPLEMENTARY TABLE 2. Hereditary cancer susceptibility panels used in the identification of germline pathogenic *ATM* variants for each patient in the case series

**Patients**	**Multi-gene hereditary cancer panel testing performed**
Patient 1	Ambry BRCA1/2 Analyses with CancerNext-Expanded + RNAinsight 67 gene, 2020<br>AIP, ALK, APC, ATM, BAP1, BARD1, BLM, BMPR1A, BRCA1, BRCA2, BRIP1, CDH1, CDK4, CDKN1B, CDKN2A, CHEK2, DICER1, FANCC, FH, FLCN, GALNT12, HOXB13, MAX, MEN1, MET, MLH1, MRE11A, MSH2, MSH6, MUTYH, NBN, NF1, NF2, PALB2, PHOX2B, PMS2, POLD1, POLE, POT1, PRKAR1A, PTCH1, PTEN, RAD50, RAD51C, RAD51D*, RB1, RET, SDHA, SDHAF2, SDHB, SDHC, SDHD, SMAD4, SMARCA4, SMARCB1, SMARCE1, STK11, SUFU, TMEM127, TP53, TSC1, TSC2, VHL and XRCC2
Patient 2	Myriad MyRisk, 28 genes, 2018<br>APC, ATM, BARD1, BMPR1A, BRCA1, BCRCA2, BRIP1, CDH1, CDK4, CDKN2A, CHEK2, EPCAM, GREM1 MLH1, MSH2, MSH6, MUTYH, NDN, PALB2, PMS2, POLE, POLD1 PTEN, RAD51C, RAD51D, SMAD4, STK11, TP53 (sequencing and deletion/duplication); MITF (sequencing only); EPCAM and GREM1
Patient 3	MSKCC Panel, 75 genes, 2017<br>ALK, APC, ATM, BAP1, BARD1, BLM, BMPR1A, BRCA1, BRCA2, BRIP1, CDH1, CDK4, CDKN2A, CHEK2, DICER1, EGFR, EPCAM, FAM175A, FH, FLCN, GATA2, GREM1, HRAS, JAK2, KIT, KRAS, MAX, MEN1, MET, MITF, MLH1, MRE11A, MSH2, MSH6, MUTYH, NBN, NF1, NF2, NRAS, PALB2, PAX5, PDGFRA, PHOX2B, PMS2, POLE, PTCH1, PTEN, RAD50, RAD51, RAD51B, RAD51C, RAD51D, RB1, RECQL4, RUNX1, SDHAF2, SDHA, SDHB, SDHC, SDHD, SMAD3, SMAD4, SMARCA4, SMARB1, STK11, SUFU, TERT, TGFBR1, TGFBR2, TMEM127, TP53, TSC1, TSC2, VHL, WT1
Patient 4	Invitae Common Hereditary Cancers Panel, Renal/Urinary Tract Cancers Panel, 56 genes, 2018<br>APC, ATM, AXIN2, BAP1, BARD1, BMPR1A, BRCA1, BRCA2, BRIP1, BUB1B, CDC73, CDH1, CDKN1C, CDKN2A (p14ARF), CDKN2A (p16INK4a), CEP57, CHEK2, DICER1, DIS3L2, EPCAM (deletion/duplication), FH, FLCN, GPC3, GREM1 (promoter region deletion/duplication), HOXB13 (c.251G>A, p.Gly84Glu variant only), KIT, MEN1, MET, MITF (c.952G>A, p.Glu318Lys variant only), MLH1, MSH2, MSH6, MUTYH, NBN, NF1, PALB2, PDGFRA, PMS2, POLD1, POLE, PTEN, RAD50, RAD51C, RAD51D, SDHA, SDHB, SDHC, SDHD, SMAD4, SMARCA4, SMARCB1, STK11, TP53, TSC1, TSC2, VHL, WT1
Patient 5	Sema4 Signal Hereditary Cancer Comprehensive Panel, 73 genes, 2021<br>AIP, ALK, APC, ATM, AXIN2, BAP1, BARD1, BLM, BMPR1A, BRCA1, BRCA2, BRIP1, CDH1, CDK4, CDKN1B, CDKN2A, CEBPA, CHEK2, DICER1, EGFR, EPCAM, FH, FLCN, GATA2, GREM1, HOXB13, HRAS, KIT,MAX, MEN1, MET, MITF, MLH1, MSH2, MSH3, MSH6, MUTYH, NBN, NF1, NF2, NTHL1, PALB2, PDGFRA, PHOX2B, PMS2, POLD1, POLE, POT1, PRKAR1A, PTCH1, PTEN,RAD51C, RAD51D, RB1, RET, RUNX1, SDHA, SDHAF2, SDHB, SDHC, SDHD, SMAD4, SMARCA4, SMARCB1, STK11, SUFU, TMEM127, TP53, TSC1, TSC2, VHL, WT1, XRCC2
Patient 6	Invitae Multi-Cancer Panel, 84 genes, 2021<br>AIP, ALK, APC, ATM, AXIN2, BAP1, BARD1, BLM, BMPR1A, BRCA1, BRCA2, BRIP1, CASR, CDC73, CDH1, CDK4, CDKN1B, CDKN1C, CDKN2A, CEBPA, CHEK2, CTNNA1, DICER1, DIS3L2, EGFR, EPCAM, FH, FLCN, GATA2, GPC3, GREM1, HOXB13, HRAS, KIT, MAX, MEN1, MET, MITF, MLH1, MSH2, MSH3, MSH6, MUTYH, NBN, NF1, NF2, NTHL1, PALB2, PDGFRA, PHOX2B, PMS2, POLD1, POLE, POT1, PRKAR1A, PTCH1, PTEN, RAD50, RAD51C, RAD51D, RB1, RECQL4, RET, RUNX1, SDHA, SDHAF2, SDHB, SDHC, SDHD, SMAD4, SMARCA4, SMARCB1, SMARCE1, STK11, SUFU, TERC, TERT, TMEM127, TP53,TSC1, TSC2, VHL, WRN, WT1

SUPPLEMENTARY TABLE 3. Characterization of variants of uncertain significance identified among the patients in case series

Patients	Data on Variants of Uncertain Significance
Patient 4	*CHEK2* c.7C>T (p.Arg3Trp), heterozygous<br><br>The patient is heterozygous for a missense variant of uncertain significance, *CHEK2* Arg3Trp. This variant has an allele count higher than expected for a pathogenic variant and while multiple functional studies have shown that this variant does not significantly affect CHEK2 activity, one study has shown that it results in a reduced response to DNA damage (10–13). The variant has been submitted to the ClinVar database twelve times since 2016 with four interpretations as "likely benign" and eight interpretations as "uncertain significance" (ID 142209). This available evidence is currently insufficient to determine its pathogenicity.
	DIS3L2 c.1447C>G (p.Arg483Gly), heterozygous<br><br>The patient is heterozygous for a missense variant of uncertain significance, *DIS3L2* Arg483Gly. This variant has previously been reported in association with a patient with a Wilms tumor, but whether the variant was somatic or of germline origin is unknown, and functional study of the variant showed that it impaired suppression of anchorage-independent cell growth in HEK293 cells (14). The variant has been submitted to the ClinVar database six times since 2016 with one interpretation as "benign" and five interpretations as "uncertain significance" (ID: 241961). This available evidence is currently insufficient to determine its pathogenicity.

SUPPLEMENTARY TABLE 4. Qualifications for hereditary cancer susceptibility testing among patients in case series based on current NCCN criteria

Patients	Qualifications for testing based on NCCN 2022.2 HBOC Guidelines for Hereditary Cancer Screening	Qualifications for testing based on NCCN 2022.1 Colorectal Guidelines for Hereditary Cancer Screening
Patient 1	Qualifies for testing based on her history of breast cancer and 1st degree relative with pancreatic cancer	
Patient 2		Qualifies for hereditary colorectal cancer testing based on personal endometrial cancer with 1st degree relative with Lynch syndrome related cancer
Patient 3	Qualifies based on 2nd degree relative with breast cancer diagnosed younger than age 46	
Patient 4		

Patient 5	Qualifies based on sarcoma present before age 45 and a 2nd degree relative with breast cancer diagnosed younger than age 46	
Patient 6		

SUPPLEMENTARY TABLE 5. Published cases of germline *ATM* variants in patients with carcinomas of the biliary tract, uterus, small bowel and ampulla, kidney, lung, and sarcoma

Cancer type	Study characteristics	Sequencing platform used	Total patients with germline pathogenic variants	Description of patients with germline *ATM* variant	Germline *ATM* variant	Citation
Biliary tract	131 patients with biliary tract cancers treated at Memorial Sloan Kettering	Illumina HiSeq2500	21 (16%)	36-year-old female with intrahepatic cholangiocarcinoma	c.3669_3670insTAG (p.Leu1224*)	(15)
	412 Japanese and Italian patients with biliary tract cancer	Illumina HiSeq 2000/2500	16 (11%)	60-year-old female with distal cholangiocarcinoma	c.7878_7882delTTATA (p.A2626fs)	(16)
	840 patients with cholangiocarcinoma	Illumina HiSeq4000	36 (4.3%)	5 patients with cholangiocarcinoma	Not provided	(17)
	60 Chinese patients with gallbladder cancer	Illumina NextSeq500	2 (3.3%)	1 patient with gallbladder adenocarcinoma	Not provided	(18)
	47 Norwegian patients with bile duct cancer	Illumina HiSeq2500/ NextSeq500	8 (17%)	1 patient with bile duct cancer	c.8833_8834del (p.Leu2945Valfs*10	(19)
Uterine	224 patients with uterine cancer who underwent multi-gene panel testing	Not provided	37 (16.5%)	61-year-old female with uterine cancer, endometrioid type	c.6679C>T, (p.R2227C)	(20)
				68-year-old female with uterine cancer, clear cell type	c.3G>A, (p.Met1?)	
	381 patients with uterine cancer treated at MD Anderson	llumina HiSeq2500	35 (9.2%)	76-year-old female with uterine cancer, endometrioid type	c.2921+1G>A	(21)
	33 patients with personal and family history of uterine cancer	Illumina NextSeq500	3 (9%)	77-year-old female with uterine cancer, endometrioid type	c.7638_7646del, (p.Arg2547_Ser2549del)	(22)
	1,542 patients with uterine cancer	Illumina HiSeq4000	76 (4.9%)	6 patients with uterine cancer	Not provided	(18)

	1,170 patients with uterine cancer treated at the Mayo clinic	Illumina HiSeq4000	53 (4.5%)	9 patients with uterine cancer	c.1564_1565delGA<br>c.1564_1565delGA<br>c.3085dupA<br>c.3245_3247delinsTGAT<br>c.6013delinsAA<br>c.6100C>T (p.Arg2034X)<br>c.8293G>A, (p.Gly2765Ser)<br>c.8766dupT<br>c.8786+1G>A	(23)
Small Bowel/ Ampulla	100 patients with small bowel carcinomas treated at Memorial Sloan Kettering	Illumina HiSeq2500	18 (18%)	2 patients with duodenal/jejunal cancer	c.278delA<br>c.7374_7375insAlu	(24)
	37 Indian patients with ampullary carcinoma	Illumina HiSeq2500	15 (40.5%)	None	None	(25)
	45 patients diagnosed with ampullary carcinoma treated at Memorial Sloan Kettering	Illumina HiSeq2500	8 (18%)	67-year-old with ampullary adenocarcinoma, pancreatobiliary type	c.8266A>T, (p.Lys2756Ter)	(26)
				69-year-old with ampullary adenocarcinoma, pancreatobiliary type	c.9023G>A, (p.Arg3008His)	
Renal Cell	322 Chinese patients diagnosed with renal cell cancer	Illumina NovaSeq6000	32 (9.9%)	82-year-old with renal cell carcinoma	c.7660del, (p.H2554Ifs*10)	(27)
				72-year-old with renal cell carcinoma	c.8494C>T (p.R2832C)	
	232 patients with early-onset renal cell carcinoma treated at Memorial Sloan Kettering	Illumina HiSeq2500	41 (17.7%)	2 patients with renal cell carcima	c.6015dupC<br>c.4236+1G>T	(28)
	254 patients with advanced renal cell carcinoma treated at Memorial Sloan Kettering	Illumina HiSeq2500	41 (16.1%)	1 patient with renal cell carcinoma	c.-30-2A>G	(29)
	294 Spanish patients with metastatic renal cell carcinoma	Illumina HiSeq	19 (6.5%)	60-year-old male with clear cell renal cell carcinoma	c.6095+1G>A	(30)

	1829 patients with renal cell carcinoma	Illumina MiSeq/ HiSeq2500	311 (17%)	16 patients with renal cell carcinoma	c.6404_6405insTT<br>c.8147T>C (p.Val2716Ala)<br>c.7705_7706delGA<br>c.1139_1142dupACAG<br>c.6146_6147delAT<br>Deletion (Exon 29)<br>c.7271T>G p.Val2424Gly<br>c.901+1G>A Splice donor<br>c.4111delG p<br>c.1179_1180delGG<br>c.2730_2731insAG<br>c.170G>A p.Trp57*<br>c.7388_7389insAlu<br>c.1139_1142dup<br>c.4761dup<br>c.5932G>T p.Glu1978*	(31)
	1336 patients with renal cell carcinoma in UK 100,000 Genomes Project	Illumina HiSeq	88 (6.6%)	10 patients with renal cell carcinoma	c.1339C>T (p.Arg447Ter)<br>c.964_968del<br>c.1442T>G (p.Leu481Ter)<br>c.1782del<br>c.2466+1G>A<br>c.3451A>T (p.Lys1151Ter)<br>c.8147T>C (p.Val2716Ala)<br>c.8147T>C (p.Val2716Ala)<br>c.652C>T (Gln218Ter)<br>c.742C>T (p.Arg248Ter)	(32)
Sarcoma	66 Asian patients with sarcoma diagnosed before age 50	Illumina HiSeq4000	9 (13.6%)	24-year-old with alveolar rhabdomyosarcoma	c.2770C>T, (p.Arg924Trp)	(33)
				49-year-old with synovial sarcoma	c.512A>G, (p.Tyr171Cys)	
	1,147 pediatric patients with pediatric sarcomas	Not provided	150 (13%)	2 pediatric patients with osteosarcoma	c.1396C>T<br>c.2251-10T>G	(34)
	1,044 patients with osteosarcoma	Illumina HiSeq2500	292 (28%)	5 patients with osteosarcoma	c.5908C>T<br>c.1564_1565del<br>c.7629+2T>C<br>c.7638_7646del<br>c.9079dupA	(35)
	394 pediatric patients with rhabdomyosarcoma	Illumina HiSeq2000	59 (15%)	Male with alveolar rhabdomyosarcoma	c.4437-1G>C	(36)
				2 pediatric patients with rhabdomyosarcoma	c.7271T>G, (p.V2424G)<br>c.8671+2T>A	
Lung	7,668 patients with advanced lung carcinoma	Illumina HiSeq4000	448 (5.8%)	60 patients with lung carcinoma	Not provided	(17)
	555 patients with lung adenocarcinoma reported in The Cancer Genome Atlas	Not provided	14 (2.5%)	52-year-old male current smoker, 36 pack years	p.E522Ifs*43	(37)
				65-year-old female former smoker, 10 pack years	p.L1764Yfs*12	
				67-year-old male current smoker, 67 pack years	p.V2716A	

				74-year-old female never smoker	p.V2716A	
				68-year-old female former smoker, 50 pack years	p.K1615Lfs*19	
				70-year-old male former smoker, 43 pack years	p.IVS51-2A>C	
				Not provided	p.S1905Ifs*25	
	5,118 patients with advanced lung cancer treated at Memorial Sloan Kettering	Illumina HiSeq2500	222 (4.3%)	26 patients with advanced lung cancer	Not provided	(38)
	1,764 Chinese lung cancer patients	Illumina NextSeq500	67 (3.8%)	66-year-old female with lung adenocarcinoma	c.497-1G>C	(39)
				51-year-old female with lung adenocarcinoma	p.D2672Ifs*8	

SUPPLEMENTARY METHODS. Tumor sequencing data for analysis of pathogenic somatic alterations were accessed online on 12/07/22 through the cBioPortal for Cancer Genomics at https://www.cbioportal.org/. Gallbladder adenocarcinoma alterations were studied by selecting Gallbladder Cancer (MSK, Cancer 2018) and Gallbladder Carcinoma (Shanghai, Nat Genet 2014). Small bowel carcinoma cases were examined by selected MSK-IMPACT Clinical Sequencing Cohort (MSK, Nat Med 2017) and MSK MetTropism (MSK, Cell 2021) and examining alterations by cancer type. Uterine carcinoma was investigated by selecting the Uterine Corpus Endometrial Carcinoma (TGCA PanCancer Atlas). Renal clear cell carcinoma was studied by selecting Kidney Renal Clear Cell Carcinoma (TCGA, PanCancer Atlas). Sarcoma was investigated by selecting Sarcoma (TGCA, PanCancer Atlas). Lung adenocarcinoma was analyzed with the Lung Adenocarcinoma (TCGA, PanCancer Atlas). Breast invasive carcinoma was studied with the Breast Invasive Carcinoma (TCGA, PanCancer Atlas). In each case, "Query by Gene" was selected and the genes *ATM*, *BRCA1*, and *CHEK2* were entered. Genomic profiling of mutations, structural variants, and putative copy-number alterations from GISTIC was selected. All samples with mutation and CNA data were chosen for analysis, and the results were further filtered to exclude all alterations of unknown significance.

The following are accession links for the somatic *ATM*, *BRCA1*, *CHEK2* alteration data found on cBioPortal. Gallbladder carcinoma: https://bit.ly/3srxbQT. Uterine carcinoma: https://bit.ly/3W3fynI. Small bowel carcinoma: https://bit.ly/3h7Zd1M. Renal cell carcinoma: https://bit.ly/3SBhUHR. Sarcoma: https://bit.ly/3gHA50V. Lung adenocarcinoma: https://bit.ly/3TTbQex. Breast adenocarcinoma: https://bit.ly/3gHyngj.

Analysis of alterations in *ATM, TP53, BRCA1,* and *CHEK2* for co-occurrence and mutual exclusivity using the pooled cancer sequencing studies can be accessed through the cBioPortal link: https://bit.ly/3j9duMe

SUPPLEMENTARY REFERENCES

1. Cavalieri S, Funaro A, Porcedda P, Turinetto V, Migone N, Gatti RA, Brusco A. ATM mutations in Italian families with ataxia telangiectasia include two distinct large genomic deletions. *Hum Mutat* (2006) 27:1061–1061. doi: 10.1002/humu.9454

2. Li A, Swift M. Mutations at the ataxia-telangiectasia locus and clinical phenotypes of A-T patients. *American Journal of Medical Genetics* (2000)

3. Frey MK, Kim SH, Bassett RY, Martineau J, Dalton E, Chern J-Y, Blank SV. Rescreening for genetic mutations using multi-gene panel testing in patients who previously underwent non-informative genetic screening. *Gynecol Oncol* (2015) 139:211–215. doi: 10.1016/j.ygyno.2015.08.006

4. Stracker TH, Roig I, Knobel PA, Marjanović M. The ATM signaling network in development and disease. *Frontiers Genetics* (2013) 4:37. doi: 10.3389/fgene.2013.00037

5. Aygün FD, Nepesov S, Çokuğraş H, Camcıoğlu Y. Bladder Wall Telangiectasia in a Patient with Ataxia-Telangiectasia and How to Manage? *Case Reports Pediatrics* (2015) 2015:615368. doi: 10.1155/2015/615368

6. Broeks A, Klein A de, Floore A, Muijtjens M, Kleijer W, Jaspers N, Veer L van 't. ATM germline mutations in classical ataxia-telangiectasia patients in the Dutch population. *Hum Mutat* (1998) 12:330–337. doi: 10.1002/(sici)1098-1004(1998)12:5<330::aid-humu6>3.0.co;2-h

7. Mitui M, Nahas SA, Du LT, Yang Z, Lai CH, Nakamura K, Arroyo S, Scott S, Purayidom A, Concannon P, et al. Functional and computational assessment of missense variants in the ataxia-telangiectasia mutated (ATM) gene: mutations with increased cancer risk. *Hum Mutat* (2009) 30:12–21. doi: 10.1002/humu.20805

8. Meissner WG, Fernet M, Couturier J, Hall J, Laugé A, Henry P, Stoppa-Lyonnet D, Tison F. Isolated generalized dystonia in biallelic missense mutations of the ATM gene. *Movement Disord* (2013) 28:1897–1899. doi: 10.1002/mds.25487

9. Carter NJ, Marshall ML, Susswein LR, Zorn KK, Hiraki S, Arvai KJ, Torene RI, McGill AK, Yackowski L, Murphy PD, et al. Germline pathogenic variants identified in women with ovarian tumors. *Gynecol Oncol* (2018) 151:481–488. doi: 10.1016/j.ygyno.2018.09.030

10. Lee SB, Kim SH, Bell DW, Wahrer DC, Schiripo TA, Jorczak MM, Sgroi DC, Garber JE, Li FP, Nichols KE, et al. Destabilization of CHK2 by a missense mutation associated with Li-Fraumeni Syndrome. *Cancer Res* (2001) 61:8062–7.

11. Delimitsou A, Fostira F, Kalfakakou D, Apostolou P, Konstantopoulou I, Kroupis C, Papavassiliou AG, Kleibl Z, Stratikos E, Voutsinas GE, et al. Functional characterization of CHEK2 variants in a Saccharomyces cerevisiae system. *Hum Mutat* (2019) 40:631–648. doi: 10.1002/humu.23728

12. Kleiblova P, Stolarova L, Krizova K, Lhota F, Hojny J, Zemankova P, Havranek O, Vocka M, Cerna M, Lhotova K, et al. Identification of deleterious germline CHEK2 mutations and their association with breast and ovarian cancer. *Int J Cancer* (2019) 145:1782–1797. doi: 10.1002/ijc.32385

13. Roeb W, Higgins J, King M-C. Response to DNA damage of CHEK2 missense mutations in familial breast cancer. *Hum Mol Genet* (2012) 21:2738–2744. doi: 10.1093/hmg/dds101

14. Astuti D, Morris MR, Cooper WN, Staals RHJ, Wake NC, Fews GA, Gill H, Gentle D, Shuib S, Ricketts CJ, et al. Germline mutations in DIS3L2 cause the Perlman syndrome of overgrowth and Wilms tumor susceptibility. *Nat Genet* (2012) 44:277–284. doi: 10.1038/ng.1071

15. Maynard H, Stadler ZK, Berger MF, Solit DB, Ly M, Lowery MA, Mandelker D, Zhang L, Jordan E, Dika IE, et al. Germline alterations in patients with biliary tract cancers: A spectrum of significant and previously underappreciated findings. *Cancer* (2020) 126:1995–2002. doi: 10.1002/cncr.32740

16. Wardell CP, Fujita M, Yamada T, Simbolo M, Fassan M, Karlic R, Polak P, Kim J, Hatanaka Y, Maejima K, et al. Genomic characterization of biliary tract cancers identifies driver genes and predisposing mutations. *J Hepatol* (2018) 68:959–969. doi: 10.1016/j.jhep.2018.01.009

17. Yap TA, Ashok A, Stoll J, Mauer E, Nepomuceno VM, Blackwell KL, Garber JE, Meric-Bernstam F. Prevalence of Germline Findings Among Tumors From Cancer Types Lacking Hereditary Testing Guidelines. *Jama Netw Open* (2022) 5:e2213070. doi: 10.1001/jamanetworkopen.2022.13070

18. Lin J, Dong K, Bai Y, Zhao S, Dong Y, Shi J, Shi W, Long J, Yang X, Wang D, et al. Precision oncology for gallbladder cancer: insights from genetic alterations and clinical practice. *Ann Transl Medicine* (2019) 7:467–467. doi: 10.21037/atm.2019.08.67

19. Bertelsen B, Tuxen IV, Yde CW, Gabrielaite M, Torp MH, Kinalis S, Oestrup O, Rohrberg K, Spangaard I, Santoni-Rugiu E, et al. High frequency of pathogenic germline variants within homologous recombination repair in patients with advanced cancer. *Npj Genom Medicine* (2019) 4:13. doi: 10.1038/s41525-019-0087-6

20. Karpel HC, Chern J-Y, J. MS, A. JS, Pothuri B. Utility of germline multi-gene panel testing in patients with endometrial cancer. *Gynecol Oncol* (2022) 165:546–551. doi: 10.1016/j.ygyno.2022.04.003

21. Ring KL, Bruegl AS, Allen BA, Elkin EP, Singh N, Hartman A-R, Daniels MS, Broaddus RR. Germline multi-gene hereditary cancer panel testing in an unselected endometrial cancer cohort. *Modern Pathol* (2016) 29:1381–1389. doi: 10.1038/modpathol.2016.135

22. Kondrashova O, Shamsani J, O'Mara TA, Newell F, Reed AEM, Lakhani SR, Kirk J, Pearson JV, Waddell N, Spurdle AB. Tumor Signature Analysis Implicates Hereditary Cancer Genes in Endometrial Cancer Development. *Cancers* (2021) 13:1762. doi: 10.3390/cancers13081762

23. Long B, Lilyquist J, Weaver A, Hu C, Gnanaolivu R, Lee KY, Hart SN, Polley EC, Bakkum-Gamez JN, Couch FJ, et al. Cancer susceptibility gene mutations in type I and II endometrial cancer. *Gynecol Oncol* (2019) 152:20–25. doi: 10.1016/j.ygyno.2018.10.019

24. Latham A, Shia J, Patel Z, Reidy-Lagunes DL, Segal NH, Yaeger R, Ganesh K, Connell L, Kemeny NE, Kelsen DP, et al. Characterization and Clinical Outcomes of DNA Mismatch Repair–deficient Small Bowel Adenocarcinoma. *Clin Cancer Res* (2021) 27:1429–1437. doi: 10.1158/1078-0432.ccr-20-2892

25. Kumari N, Singh RK, Mishra SK, L R, Mohindra S, Krishnani N. Prevalence and spectrum of pathogenic germline variants in intestinal and pancreatobiliary type of ampullary cancer. *Pathology - Res Pract* (2021) 217:153309. doi: 10.1016/j.prp.2020.153309

26. Wong W, Lowery MA, Berger MF, Kemel Y, Taylor B, Zehir A, Srinivasan P, Bandlamudi C, Chou J, Capanu M, et al. Ampullary cancer: Evaluation of somatic and germline genetic alterations and association with clinical outcomes. *Cancer* (2019) 125:1441–1448. doi: 10.1002/cncr.31951

27. Kong W, Yang T, Wen X, Mu Z, Zhao C, Han S, Tian J, Zhang X, Zhou T, Zhang Y, et al. Germline Mutation Landscape and Associated Clinical Characteristics in Chinese Patients With Renal Cell Carcinoma. *Frontiers Oncol* (2021) 11:737547. doi: 10.3389/fonc.2021.737547

28. Truong H, Sheikh R, Kotecha R, Kemel Y, Reisz PA, Lenis AT, Mehta NN, Khurram A, Joseph V, Mandelker D, et al. Germline Variants Identified in Patients with Early-onset Renal Cell Carcinoma Referred for Germline Genetic Testing. *European Urology Oncol* (2021) 4:993–1000. doi: 10.1016/j.euo.2021.09.005

29. Carlo MI, Mukherjee S, Mandelker D, Vijai J, Kemel Y, Zhang L, Knezevic A, Patil S, Ceyhan-Birsoy O, Huang K-C, et al. Prevalence of Germline Mutations in Cancer Susceptibility Genes in Patients With Advanced Renal Cell Carcinoma. *Jama Oncol* (2018) 4:1228. doi: 10.1001/jamaoncol.2018.1986

30. Santos M, Lanillos J, Roldan-Romero JM, Caleiras E, Montero-Conde C, Cascón A, Climent MA, Anguera G, Hernando S, Laínez N, et al. Prevalence of pathogenic germline variants in patients with metastatic renal cell carcinoma. *Genet Med* (2021) 23:698–704. doi: 10.1038/s41436-020-01062-0

31. Alaiwi SA, Nassar AH, Adib E, Groha SM, Akl EW, McGregor BA, Esplin ED, Yang S, Hatchell K, Fusaro V, et al. Trans-ethnic variation in germline variants of patients with renal cell carcinoma. *Cell Reports* (2021) 34:108926. doi: 10.1016/j.celrep.2021.108926

32. Yngvadottir B, Andreou A, Bassaganyas L, Larionov A, Cornish AJ, Chubb D, Saunders CN, Smith P, Zhang H, Cole Y, et al. Frequency of pathogenic germline variants in cancer susceptibility genes in 1336 renal cell carcinoma cases. *Hum Mol Genet* (2022) 31:ddac089. doi: 10.1093/hmg/ddac089

33. Chan SH, Lim WK, Ishak NDB, Li S-T, Goh WL, Tan GS, Lim KH, Teo M, Young CNC, Malik S, et al. Germline Mutations in Cancer Predisposition Genes are Frequent in Sporadic Sarcomas. *Sci Rep-uk* (2017) 7:10660. doi: 10.1038/s41598-017-10333-x

34. Gillani R, Camp SY, Han S, Jones JK, Chu H, O'Brien S, Young EL, Hayes L, Mitchell G, Fowler T, et al. Germline predisposition to pediatric Ewing sarcoma is characterized by inherited pathogenic variants in DNA damage repair genes. *Am J Hum Genet* (2022) 109:1026–1037. doi: 10.1016/j.ajhg.2022.04.007

35. Mirabello L, Zhu B, Koster R, Karlins E, Dean M, Yeager M, Gianferante M, Spector LG, Morton LM, Karyadi D, et al. Frequency of Pathogenic Germline Variants in Cancer-Susceptibility Genes in Patients With Osteosarcoma. *Jama Oncol* (2020) 6:724–734. doi: 10.1001/jamaoncol.2020.0197

36. Kim J, Light N, Subasri V, Young EL, Wegman-Ostrosky T, Barkauskas DA, Hall D, Lupo PJ, Patidar R, Maese LD, et al. Pathogenic Germline Variants in Cancer Susceptibility Genes in Children and Young Adults With Rhabdomyosarcoma. *Jco Precis Oncol* (2021) 5:75–87. doi: 10.1200/po.20.00218

37. Parry EM, Gable DL, Stanley SE, Khalil SE, Antonescu V, Florea L, Armanios M. Germline Mutations in DNA Repair Genes in Lung Adenocarcinoma. *J Thorac Oncol* (2017) 12:1673–1678. doi: 10.1016/j.jtho.2017.08.011

38. Mukherjee S, Bandlamudi C, Hellmann MD, Kemel Y, Drill E, Rizvi H, Tkachuk K, Khurram A, Walsh MF, Zauderer MG, et al. Germline Pathogenic Variants Impact Clinicopathology of Advanced Lung Cancer. *Cancer Epidemiology Biomarkers Prev* (2022) 31:1450–1459. doi: 10.1158/1055-9965.epi-21-1287

39. Tian P, Cheng X, Zhao Z, Zhang Y, Bao C, Wang Y, Cai S, Ma G, Huang Y. Spectrum of Pathogenic Germline Mutations in Chinese Lung Cancer Patients through Next-Generation Sequencing. *Pathol Oncol Res* (2020) 26:109–114. doi: 10.1007/s12253-019-00771-5
